# Supplementary material for: Integrated analysis of the aqueous humor microbiome and lens capsule transcriptome in high myopia cataract: a pilot study
Source: Front Med (Lausanne). 2026 Jun 16;13:1845205. doi: 10.3389/fmed.2026.1845205 (PMC13314463; doi:10.3389/fmed.2026.1845205)
Supplement: Supplementary file 3 [file Data_Sheet_1.zip › 4.Meta_Statistic/C372089/LEfSe/LEfSe_table.res.pdf]

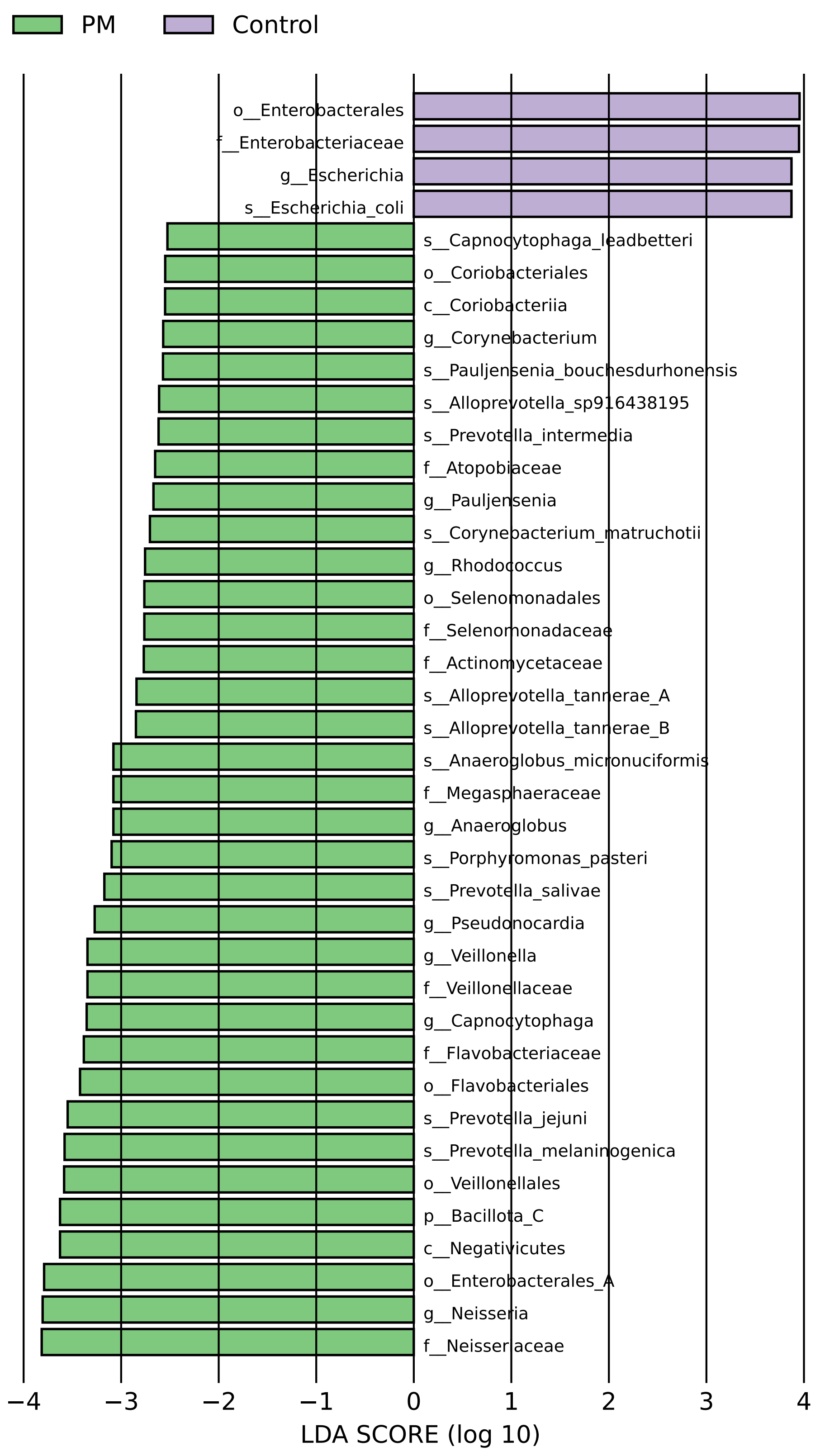
PM
Control
o__Enterobacterales
f__Enterobacteriaceae
g__Escherichia
s__Escherichia_coli
s__Capnocytophaga_leadbetteri
o__Coriobacteriales
c__Coriobacteriia
g__Corynebacterium
s__Pauljensenia_bouchesdurhonensis
s__Alloprevotella_sp916438195
s__Prevotella_intermedia
f__Atopobiaceae
g__Pauljensenia
s__Corynebacterium_matruchotii
g__Rhodococcus
o__Selenomonadales
f__Selenomonadaceae
f__Actinomycetaceae
s__Alloprevotella_tannerae_A
s__Alloprevotella_tannerae_B
s__Anaeroglobus_micronuciformis
f__Megasphaeraceae
g__Anaeroglobus
s__Porphyromonas_pasteri
s__Prevotella_salivae
g__Pseudonocardia
g__Veillonella
f__Veillonellaceae
g__Capnocytophaga
f__Flavobacteriaceae
o__Flavobacteriales
s__Prevotella_jejuni
s__Prevotella_melaninogenica
o__Veillonellales
p__Bacillota_C
c__Negativicutes
o__Enterobacterales_A
g__Neisseria
f__Neisseriaceae
−4
−3
−2
−1
0
1
2
3
4
LDA SCORE (log 10)
